# Fostering Peat Moss Feedbacks to Accelerate Peatland Restoration

**DOI:** 10.1111/gcb.70788

**Published:** 2026-03-25

**Authors:** Ralph J. M. Temmink, Benjamin M. Delory, Max Rietkerk, Alfons J. P. Smolders, Leon P. M. Lamers, Avni Malhotra, Line Rochefort, Gustaf Granath, John Couwenberg, Gerbrand Koren, Bjorn J. M. Robroek

**Affiliations:** ^1^ Copernicus Institute of Sustainable Development Utrecht University Utrecht the Netherlands; ^2^ Department of Ecology, Radboud Institute for Biological and Environmental Sciences Radboud University Nijmegen the Netherlands; ^3^ B‐WARE Research Centre Nijmegen the Netherlands; ^4^ Biological Sciences Division Pacific Northwest National Laboratory Richland Washington USA; ^5^ Peatland Ecology Research Group, Centre for Northern Studies Université Laval Quebec Canada; ^6^ Department of Ecology and Genetics Uppsala University Uppsala Sweden; ^7^ Institute of Botany and Landscape Ecology University of Greifswald, Partner in the Greifswald Mire Centre Greifswald Germany; ^8^ School of Biological Sciences, Faculty of Environmental and Life Sciences University of Southampton Southampton UK

**Keywords:** bog, facilitation, feedbacks, fen, habitat modification, peat moss, review, *Sphagnum*, spontaneous regeneration

## Abstract

Extensive knowledge exists on plant‐species traits and functions, but we understand less about how population‐ or community‐level emergent traits influence ecosystem functioning. This knowledge gap is important for ecosystems like peatlands, arid drylands, salt marshes, seagrass meadows, and mangroves, where emergent traits of plant communities can create plant‐environment feedbacks that amplify or dampen ecosystem processes. Recent insights from restoration ecology suggest that these feedbacks can critically influence restoration success. Despite growing recognition of emergent trait‐driven feedbacks in other ecosystems, they remain underexplored in peatland restoration, the world's most carbon‐dense ecosystem. Here, we review emergent self‐amplifying and self‐dampening feedbacks with net positive effects for peat moss‐dominated systems. We show how these feedbacks can promote key physical, chemical, and biological processes that enhance peat moss growth, increase water retention, and reduce microbial decomposition of organic matter. Understanding and fostering these feedbacks offers a promising framework to accelerate peatland restoration across diverse degradation states.

## Graphical abstract

Alternative stable states and peat moss and vascular plant feedbacks in peatlands. Selected feedbacks in a peat moss‐dominated state or a vascular plant‐dominated state. The two insets show ecosystem states (dot) in resilience landscapes (bottom). The plus symbol (+) indicates a self‐amplifying feedback, while a minus symbol (−) indicates a self‐dampening feedback.

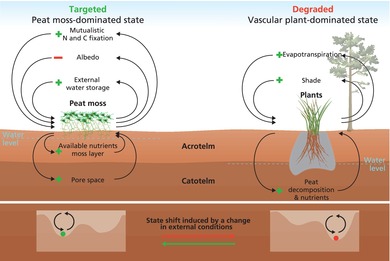

## 1 Introduction

**FIGURE 1:**
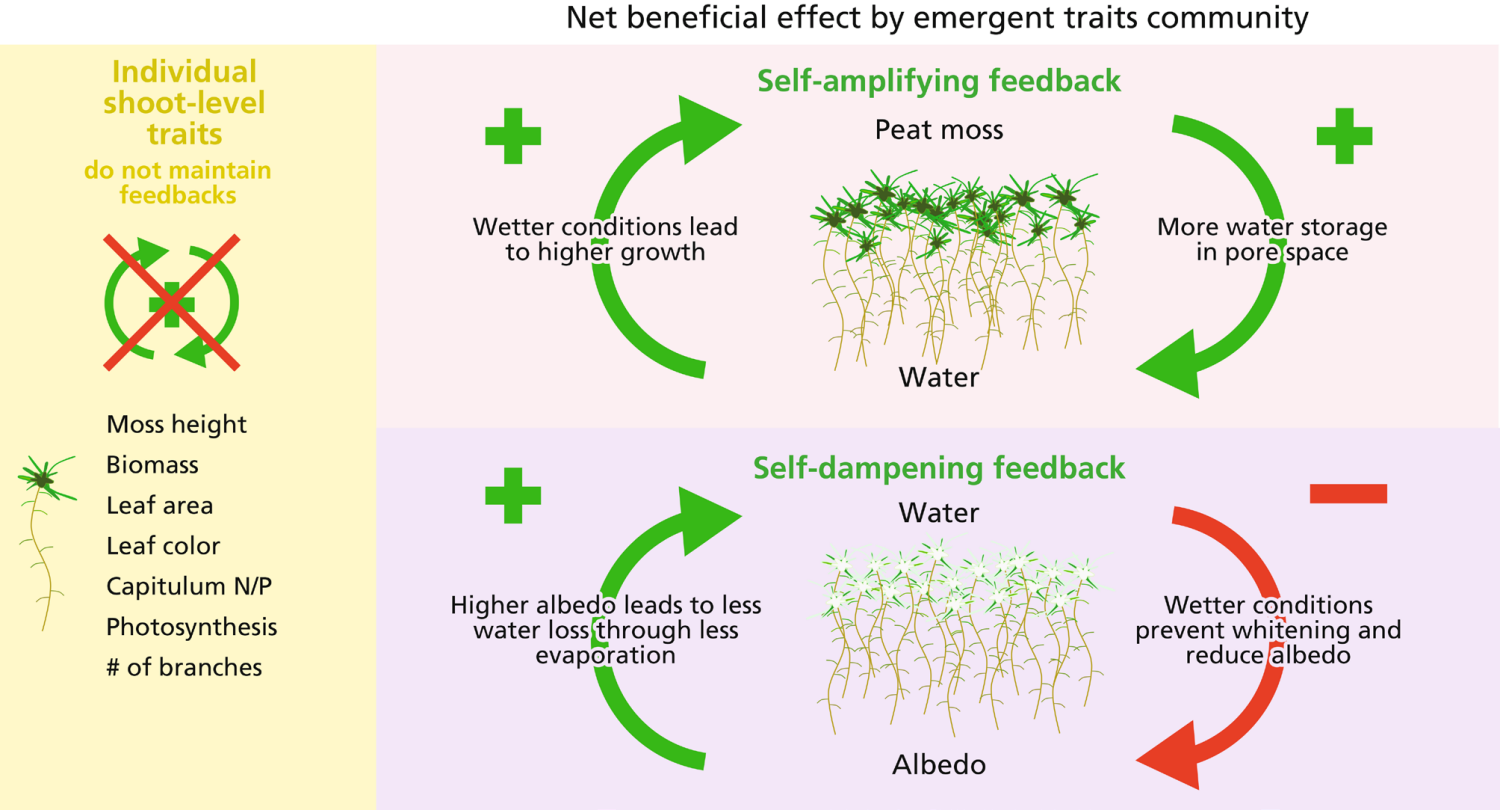
Examples of emergent peat moss feedbacks. Peat mosses modify their environment through feedbacks generated by emergent traits at larger aggregate scales. The mechanism can already function at the individual shoot level, but the beneficial effect of emergent feedbacks is larger than that at the individual level; i.e., community level. Both self‐amplifying and self‐dampening feedbacks result in a net beneficial effect on peat moss growth and stabilization. The plus symbol (+) indicates an effect in the same direction (wetter becomes wetter, named moss productivity feedback) (Waddington et al. 2015), while a minus symbol (−) indicates an effect in the opposite direction (drought dries the environment, increased albedo maintains a wet environment, named moss surface resistance and albedo feedback) (Waddington et al. 2015).

Ecosystems like peatlands, salt marshes, mangroves, arid drylands and seagrass meadows are often dominated by habitat‐modifying plants, also known as ecosystem engineers (Jones et al. 1994). Habitat modifiers can change their environment to better suit their own needs. These changes commonly rely on self‐amplifying feedbacks (i.e., positive feedbacks, Figure 1). After establishment, habitat modifiers can stabilize their environment through self‐dampening feedbacks (i.e., negative feedbacks or self‐regulation, Figure 1). Many of these feedbacks are grounded in emergent traits, which are traits that are not expressed by individual organisms, but by populations or communities at a range of organizational levels or spatial scales (Jablonski 2008; Temmink et al. 2020). Feedbacks that result from such emergent traits, hereafter emergent feedbacks, often have a net beneficial effect on the individual habitat modifying organisms (i.e., self‐facilitation) and are often stronger in larger patches and higher densities than in small patches or for individuals (Bouma et al. 2009; Maxwell et al. 2016; Robroek et al. 2009; Silliman et al. 2015). For example, emergent feedbacks in aquatic habitats can occur when a patch of macrophytes traps sediment and improves water clarity. Improved water clarity then enhances macrophyte growth due to more light availability to the plant community (Lawson et al. 2012; Maxwell et al. 2016). Under harsh environmental conditions, such as nutrient scarcity, high or variable moisture levels, or unstable sediments, growth and survival of the habitat modifier is only possible if density and patch size reach a critical mass. Hence, survival or establishment is limited when patch size remains too small for the emergence of feedbacks to modify the environment (Silliman et al. 2015).

Emergent feedbacks play a critical role in the functioning and stability of ecosystems shaped by habitat‐modifying plants (Bruno et al. 2003; DeAngelis et al. 2012; Silliman et al. 2024). The importance of emergent feedbacks in restoration is increasingly recognised, especially in coastal systems (Maxwell et al. 2016; Silliman et al. 2015, 2024; Temmink, Angelini, et al. 2023; Temmink et al. 2020, 2022; van Katwijk et al. 2016). In salt marshes, for example, simple clumping of cordgrass transplants instead of classically applied forestry‐style dispersed planting resulted in a doubling of overall restoration success (Silliman et al. 2015). Similarly, artificial structures that mimic dense patches of stiff stem canopies greatly enhance survival and growth of cordgrass transplants (Temmink et al. 2020). Consequently, researchers and practitioners are increasingly working towards integrating these feedbacks into ecological restoration, aligning with broader global restoration efforts that are driven by initiatives such as the UN Decade on Ecosystem Restoration, EU Nature Restoration Regulation and the Paris Climate Agreement (Convention on Wetlands 2021).

Among ecosystems targeted by global conservation and restoration initiatives, peatland ecosystems are particularly important because they provide large ecosystem carbon stocks with unparalleled carbon densities (Temmink et al. 2022; Xu et al. 2018). Peatlands also provide other ecosystem services, including freshwater and nutrient retention, food, medicinal plants, the support of specialized biodiversity as well as paleo‐archives (Bonn et al. 2016; Jurasinski et al. 2020; Zedler and Kercher 2005). Despite these important services, globally about 57 million hectares (ha) of peatlands are currently drained and degrading (UNEP 2022). This results in high carbon emissions (4%–5% of global human‐induced emissions are from degraded peatland on circa 0.3% of the land), land subsidence, decreased water safety and the loss of biodiversity (Temmink, Robroek, et al. 2023; UNEP 2022). This makes the restoration of peatlands urgent worldwide (Fluet‐Chouinard et al. 2023; Leifeld and Menichetti 2018; UNEP 2022). Accelerating peatland restoration requires a clear understanding of emergent feedbacks in peat moss–dominated ecosystems, like bogs and poor fens, because these feedbacks underlie the regulation of the functioning of these ecosystems. Currently, knowledge of ecological peatland feedbacks has not yet been synthesized in the context of peatland restoration.

Peatland restoration practices have largely focussed on hydrology to halt the degradation of peatlands (Convention on Wetlands 2021; Evans et al. 2021; Günther et al. 2020). However, hydrological restoration often does not result in a rapid (< 25 years) recovery of habitat modifiers and the emergent feedbacks they generate (Allan et al. 2023; Klimkowska et al. 2019; Kreyling et al. 2021). Yet, peatland restoration has, over the past decades, seen advancements including surface layer transplantation (vegetation) or inoculation (Allan et al. 2023; González and Rochefort 2014; Shepherd et al. 2023), re‐establishment of peat moss at larger spatial scales (Breton et al. 2026; Gaudig et al. 2018; Temmink et al. 2024) and the recovery of characteristic hummock‐hollow patterns (Pouliot et al. 2011). Still, the use of such restoration measures has been relatively limited compared to the extent of degradation, and there is potential to more effectively restore degraded peatlands by a wider adoption of measures beyond hydrological modification.

Peat mosses (*Sphagnum* spp.) are a classic and spatially‐dominant habitat modifier in temperate and boreal peatlands (Norby et al. 2019; Rochefort 2000; Van Breemen 1995). In these biomes, peat moss occurs both in bogs, where the vegetation is fed by rain, and in fens, where vegetation is also fed by groundwater (Rydin and Jeglum 2013). As peat moss species have no roots, stomata or vascular systems to conduct and take up water and transpire, they are susceptible to desiccation (Keane et al. 2025; Rice et al. 2008). However, (emergent) moss‐environment feedbacks keep the mosses sufficiently wet to sustain growth through enhanced water holding capacity and increased capillary rise (Clymo and Hayward 1982; Kuuri‐Riutta et al. 2024; Malhotra et al. 2016; Robroek et al. 2009; Waddington et al. 2015). Moreover, the strength of the feedbacks depends on which species are present because of different species' traits and on the variation within species (Bengtsson et al. 2016, 2020; Johnson et al. 1990). Feedback mechanisms in peat moss vegetations can act at different scales: from individual mosses (Rydin 1985), through communities (Robroek et al. 2009), and ecosystems (Couwenberg et al. 2022). They also operate over various time periods, from almost immediate to months to several centuries. Overall, the feedback mechanisms generally lead to emergent feedbacks with a net beneficial effect on peat moss growth (Couwenberg et al. 2022; Waddington et al. 2015) and result in a peat moss‐dominated state. However, when the ecosystem dries or becomes more nutrient‐rich (eutrophication), these feedbacks weaken, allowing faster‐growing vascular plants to outcompete peat moss, resulting in a vascular‐plant dominated system. It is known that when vascular plants dominated the vegetation community of a bog or fen peatland, its peat accumulation potential diminishes (Rydin et al. 2006; Berube and Rochefort 2018; Beaulne et al. 2021).

Here, we synthesize the role of emergent feedbacks on ecosystem processes in the context of ecosystem restoration in peat moss‐dominated ecosystems like bogs and open poor fens. Because many processes or feedbacks have been studied in isolation with results scattered in the literature, we undertake a first effort to synthesize these findings, providing an overview of feedbacks to provide guidance for ecological restoration. We highlight the breakdown of feedbacks under global change and show how to utilize peat moss feedbacks and disrupt vascular‐plant feedbacks to accelerate peatland restoration. Peatland restoration will benefit from emphasis on emergent feedbacks in restoration design. Furthermore, the concepts presented here can be translated to other feedback‐driven ecosystems, and thus help advance restoration science as a whole.

## 2 Emergent Feedbacks in Peat Moss‐Dominated Peatlands

**FIGURE 2:**
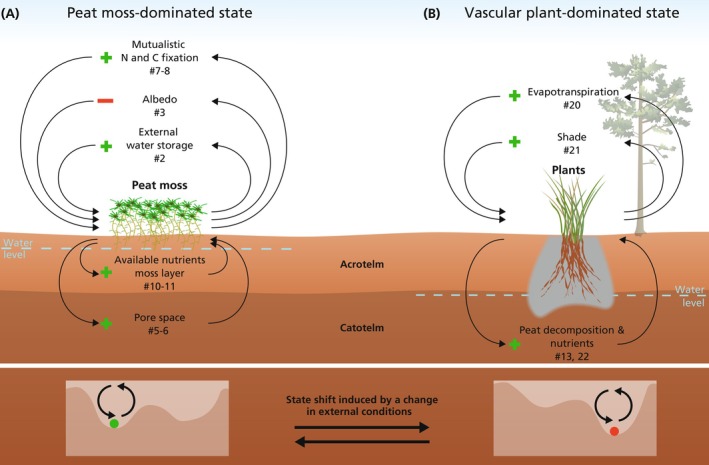
Alternative stable states and peat moss and vascular plant feedbacks in peatlands. Selected feedbacks in a peat moss‐dominated state (A) or a vascular plant‐dominated state (B). Arrows indicate feedback mechanisms in peat systems, which may also represent changes in competition or interaction dynamics (for details see Tables 1 and 2). Two insets showing ecosystem state (dot) in a resilience landscape (bottom). The shaded part (grey) in B depicts an oxidized area through radial oxygen loss. The plus symbol (+) indicates a self‐amplifying feedback, while a minus symbol (−) indicates a self‐dampening feedback. The feedbacks shown are not exhaustive and have an illustrative purpose (for additional feedbacks and the explanation of each numbered feedback see Tables 1 and 2).

*Sphagnum*‐dominated peatlands show a range of feedback mechanisms that operate across spatial and temporal scales (Table 1). Feedbacks can be self‐dampening or self‐amplifying. Amplifying or positive feedbacks reinforce peat moss dominance by enhancing its resilience and competitive advantage over vascular plants, contributing to the persistence of a peat moss‐dominated state (Figure 2). We classify feedbacks into three functional categories: physical, chemical, and biological. Physical feedbacks include hydrological processes, such as peat compaction under low water tables, which increases capillarity and improves water availability for peat moss. We do not address hydrological feedbacks that regulate the lateral flow of water (see for example Couwenberg et al. 2022; Waddington et al. 2015). Chemical feedbacks include peat moss decomposition by‐products (organic acids) that acidify the environment, reducing vascular plant growth and microbial decomposition and further promoting peat moss. Biological feedbacks involve structural traits, such as the moss's water‐holding capacity and morphological plasticity, that increase drought resilience and promote vertical growth. Physical, chemical, and biological feedbacks often interact and sometimes within the same feedback. For example, capillarity (physical) enhances water availability, which supports growth adaptations (biological), while low nutrient availability and acidification (chemical) suppress competitors. Together, these mechanisms contribute to the formation of a highly resilient and self‐sustaining peat moss‐dominated ecosystem. Detailed descriptions and references for each feedback are presented in Table 1.

**TABLE 1:** Feedbacks in peat moss‐dominated systems.

Feedback name	Feedback type	Feedback description	Restoration potential and temporal scale to build the feedback			Operates under the following conditions		References
			Restoration potential	Practice	Temporal scale to build the feedback	Conditions	Spatial scale	
*Physical feedbacks that increase peat moss's competitive edge*								
(1) Mechanical plant or peat porosity (self‐dampening)	Physical	At lower groundwater levels during drought, peat moss and peat is compacted, which results in a denser structure that lowers hydraulic conductivity, decreasing pore size, and via higher capillarity, leading to more water availability that counteracts drought effects.	Low	Hard to manipulate as a thick layer of peat moss is often not available	Years to decades	All	mm^2^ to m^2^	Clymo and Hayward (1982); Couwenberg et al. (2022); McCarter and Price (2014); Price (1997); Price and Schlotzhauer (1999); Waddington et al. (2015)
(2) External water storage (self‐amplifying)	Physical	Larger peat moss patches hold water in external capillary spaces (between stems/leaves), which maintains more favourable peat moss growth conditions and results in higher water holding.	Medium	Introducing large and high‐density peat moss patches or apply the moss layer transfer technique	Years	All	cm^2^ to m^2^	Bengtsson et al. (2020); Gage et al. (2024); Ingram (1982); Joosten (1993); Robroek et al. (2009); Rydin (1993)
(3) Albedo (self‐dampening)	Physical	During drought, peat moss capitula whitens as the cells fill with air, which increases albedo and reduces evaporative losses, maintaining humid conditions below the capitula, thus counteracting drought effects and preventing die‐off.	High	Introduction of peat moss, reduce evaporation by addition of straw	Years	All	cm^2^ to m^2^	Clymo and Hayward (1982); Harris (2008); Price et al. (1998); Waddington et al. (2015)
(4) Temperature (self‐amplifying)	Physical	*Sphagnum* peat conducts heat poorly resulting in lower peat temperatures (i.e., below the mosses), shortening the growing season for vascular plants, which lowers competition and favors peat moss growth resulting in more peat moss peat that maintains lower temperatures. The top 5 cm of the peat moss lawn is relatively warm, stimulating peat moss growth.	Low	Addition of straw; Hard to alter temperature	Years to decades	Boreal	m^2^ to ha	Clymo and Hayward (1982); Price et al. (2003); Van Breemen (1995)
(5) Pore space: storativity (self‐amplifying)	Physical	Peat moss decomposition leads to a loss of pore space and a denser top peat layer (storativity; high peat bulk density as a proxy, small pores). Loss of pore space reduces water storage capacity, but increases the proportion of small pores, which hold water more tightly due to higher capillary forces, and can improve water availability during dry periods.	Medium	Newly formed peat moss carpets post restoration are not as dense and heavy machinery can be used to densify the acrotelm layer	Decades to centuries	All	dm^2^ to m^2^	Bu et al. (2013); Dau (1821); Gauthier et al. (2018); Gauthier et al. (2022); Johnson et al. (1990); McCarter and Price (2015); Rydin (1995); Waddington et al. (2015)
(6) Pore space: transmissivity (self‐amplifying)	Physical	In the top layer of fresh peat with large pores (transmissivity; low peat bulk density as a proxy, large pores) water storage capacity and transmissivity are higher, that prevents waterlogging while maintaining a larger reservoir of water, creating optimal growth conditions for peat mosses.	Medium to low	Peat inversion, the in situ flipping of degraded peat using a backhoe to decompact and increase pore space. Hard to manipulate: introducing peat moss to form a high porosity layer	Decades to centuries	All	dm^2^ to m^2^	Bu et al. (2013); Dau (1821); Gauthier et al. (2018); Gauthier et al. (2022); Johnson et al. (1990); McCarter and Price (2015); Rydin (1995); Waddington et al. (2015)
*Chemical feedbacks that increase peat moss's competitive edge*								
(7) Methane oxidizers presence (self‐amplifying)	Chemical	Methane‐oxidizing bacteria in peat moss provide carbon to the peat moss that grow floating in (CO_2_‐limited) water, which improves peat moss growth and provides more space for oxidizing bacteria.	Low	The presence of methane‐oxidizing bacteria can be stimulated by introducing peat moss and maintaining a very highwater level to form floating mats	Days to weeks	Carbon‐limited waters	< mm^2^	Kip et al. (2010, 2012); Raghoebarsing et al. (2005); Sundh et al. (1995)
(8) Nitrogen‐fixing microbes presence (self‐amplifying)	Chemical	Activity of diazotrophic microorganisms (e.g., cyanobacteria and methanotrophs) that live on the surface and inside dead hyaline cells fix N_2_ gas, which improves N availability for peat moss, favours its growth, and creates more space for the microorganisms that results in higher activity.	Low	Hard to manipulate	Days	Nitrogen‐limited bogs and fens	< mm^2^	Larmola et al. (2014); Raghoebarsing et al. (2005); van den Elzen et al. (2020); Vile et al. (2014)
(9) Acidification (self‐amplifying)	Chemical	Peat moss forms an acidic organic layer, which decomposes and releases organic acids into the free water, which creates conditions that reduces vascular plant growth and lowers competition, thus increasing peat moss growth, which leads to more acidification. Acidification by peat moss lowers pH of the water, which lowers concentrations of the toxic bicarbonate (HCO_3_^−^) and results in more vital mosses that can further acidify the environment.	High	Actively adding acidic substances to acidify the surface or irrigation water (e.g., hydrochloric acid) or I introducing peat moss	Days to decades	All Presence of bicarbonate‐rich water	> mm^2^	Koks et al. (2019, 2022, 2025); Soudzilovskaia et al. (2010); Vicherová et al. (2017); Wieder and Vitt (2006)
(10) Effective nutrient uptake (self‐amplifying)	Chemical	Peat moss effectively takes up and recycles (micro)nutrients (N, K, Fe, C), which lowers their availability for vascular plants. This reduces competition, leading to higher peat moss growth that effectively takes up and recycles nutrients.	Medium	Introducing peat moss. Restoring nutrient poor conditions with topsoil removal	Years to decades	All	dm^2^ to m^2^	Fritz et al. (2014); Malmer et al. (1994); Smolders et al. (2001)
(11) Nitrogen filter (self‐amplifying)	Chemical	Peat moss carpets efficiently take up N from atmospheric deposition, but also prevent N leaching to the rhizosphere of vascular plants, which impedes vascular plant growth and reduces competitive pressure leading to higher peat moss growth that captures N.	Medium	Introducing peat moss but success depends on external N deposition	Years to decades	Low to medium nitrogen deposition (up to 10 kg ha^−1^ year^−1^)	> m^2^	Chiwa et al. (2016); Lamers et al. (2000); Limpens et al. (2011); Malmer et al. (1994); Moore et al. (2005)
*Biological and biophysical feedbacks that increase peat moss' competitive edge*								
(12) Internal water storage (self‐amplifying)	Biological	Peat moss stores water in hyaline cells. By increasing density and patch size, water storage capacity of the system increases, which acts as a buffer during dry periods, promoting photosynthesis and growth, and leading to more water storage.	Medium	Introducing peat moss species from the *Acutifolia* or *Sphagnum* sub‐genus (i.e., abundant peat building peat moss of your region)	Days	All	> mm^2^	Bu et al. (2013); Chirino et al. (2006); Clymo and Hayward (1982); Hájek and Beckett (2008); Quinty et al. (2020); Silvola (1990)
(13) Peat formation (self‐amplifying)	Biological	Low decomposition rates of recalcitrant peat moss matter facilitate organic matter buildup, resulting in a living peat moss layer with greater distance to the groundwater (often HCO_3_^−^ rich), which results in ombrotrophication (i.e., a peatland to become ombrotrophic, mostly rainwater fed) that promotes peat moss growth.	High	Stimulating peat formation by water level regulation (stable optimal water levels, avoid long‐term flooding) and tree removal	Decades to centuries	All	> mm^2^ to > m^2^	Granath et al. (2010); Mettrop et al. (2014); van Bergen et al. (2020); Verhoeven and Liefveld (1997)
(14) Intraspecific trait plasticity (self‐amplifying)	Biological	Peat mosses change their growth form (morphology and architecture). For example, during repeated drought, apical dominance is suppressed, and peat mosses grows more branches, resulting in a denser structure, increasing capillarity resulting in higher water availability that favours moss growth. Under wet conditions, the opposite occurs.	Medium	Water level regulation and the introducing moss species from the *Acutifolia* sub‐genus (i.e., hummock‐forming)	Years to decades	Under long(er) term drought or wetness	mm^2^ to m^2^	Bengtsson et al. (2021); Couwenberg et al. (2022); Grau‐Andrés et al. (2022); Green (1968); Masing (1984); Oke et al. (2020); Smolyanitsky (1977)
(15) Floatability (self‐amplifying)	Biophysical	Dense mats of submerged‐growing peat moss (or floating peat) trap CH_4_, which increases floatability, improving conditions for photosynthesis, which stimulates growth leading to denser mats that better trap CH_4_.	High	Application of temporal biodegradable structures that float and support peat moss or dead wood, can be combined with introducing peat moss	Years	Open water with submerged peat moss growth	> dm^2^	Smolders et al. (2002, 2003); Temmink, Cruijsen, Smolders et al. (2021); Tomassen, Smolders, Lamers et al. (2004)
(16) Microclimate (self‐amplifying)	Biophysical	*Initiation of peat moss feedbacks*: Bryophytes (particularly *Polytrichum* species) create moist microenvironments protecting against frost heaving (the upward swelling of soil due to the freezing of water within it), and creating safe sites for peat moss spore germination, establishment and growth. However, in groundwater‐influenced sites, frost heaving can lift mosses out of the toxic bicarbonate‐rich zone, which leads to drier mosses that results in less groundwater influence and better moss growth.	High	Use the moss layer transfer technique by introducing peat moss in association with *Polytrichum* mosses (in combination and at the same time)	Years	All	> dm^2^	Buttler et al. (1998); Groeneveld and Rochefort (2005); Groeneveld et al. (2007); Grosvernier et al. (1995); Price et al. (1998); Price (1996); Quinty et al. (2020); Rochefort (2000); Słowińska et al. (2022)
(17) Vascular plant germination (self‐amplifying)	Biological	Dense peat moss cover inhibits germination and establishment of vascular plants, leading to reduced competitive pressure by vascular plants and results in higher moss growth that more effectively lowers germination of plants.	Medium	Introducing peat moss and controlling vascular plants	Years to decades	All	dm^2^ to m^2^	Boatman (1962); Bourgeois et al. (2012)
(18) Physical support (self‐amplifying)	Biophysical	*Initiation of peat moss feedbacks* via *interspecific facilitation*: Low densities of specific vascular plants, shrubs, and their roots provide physical support for establishing and stabilizing peat moss hummocks, and preventing runoff erosion, which facilitates vertical growth of hummocks.	High	Co‐transplant/seed vascular plants that can provide physical support (e.g., ericoids) combined with introducing peat moss	Years	All	> dm^2^ to m^2^	Keightley et al. (2023); Malmer et al. (1994); Osvald (1923); Pouliot et al. (2011); Telgenkamp et al. (2025); Weber (1902)
(19) Herbivory prevention (self‐amplifying)	Biological	Peat moss is an unsuitable food source for herbivores, which results in low grazing pressure, which stimulates survival and growth. The absence of birds and trees results in a low nutrient input rate via feces, which maintains the ombrotrophic state and favors the dominance of peat mosses.	Low	Lowering herbivory is typically not needed because of low grazing pressure on peat mosses	Days	All	> mm^2^	Chen et al. (2021, 2024); Mendes et al. (2020); Tomassen et al. (2005); Verhoeven and Liefveld (1997)

*Note:* A prerequisite to establish *Sphagnum* feedbacks and disrupt vascular‐plants feedbacks is that the site is rewetted. The feedbacks are ordered according to the dominant feedback type (physical, chemical, biological) and according to their spatial scale (small to large). The temporal scale to build feedbacks describes how long it takes for feedbacks to emerge, not how fast they respond to environmental changes. Self‐amplifying feedbacks are indicated with a dark shade color, where peat moss promotes conditions that further increase peat moss's development until carrying capacity is reached or poor environmental conditions overwhelm the feedback. Self‐dampening feedbacks are indicated with a light shade color, where conditions are maintained by feedback loops that slow or stop change. The literature list is not exhaustive. The table was compiled through a combination of literature review and expert judgement. Restoration potential, practice, and temporal scale to build the feedback were assessed using expert judgement informed by published restoration studies, observed recovery trajectories, ecological constraints, and the technical feasibility of restoration measures described in the literature.

## 3 Global Change: Disruption of Peat Moss Feedbacks

### 3.1 Environmental Change

**FIGURE 3:**
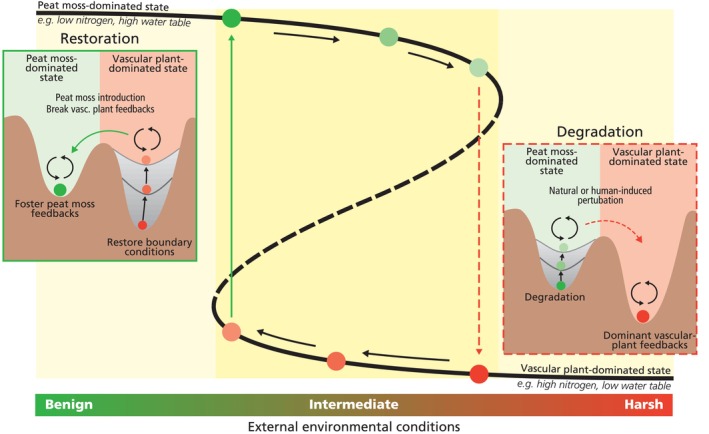
Conceptual overview showing two alternative stable states across a stress gradient of external environmental conditions with corresponding resilience landscapes. Under intermediate harsh conditions (e.g., drainage or nitrogen deposition), high peat moss density and large patch sizes can generate emergent traits that locally buffer environmental stress. This self‐facilitation enhances growth and survival once a critical threshold is surpassed (black dotted line) but makes natural recovery and restoration challenging below that threshold. Restoration can be improved by explicitly either disrupting vascular plants and fostering peat moss feedbacks (left inset, green arrow). This concept follows alternative stable state theory and hysteresis. Insets: The system shows two basins of attraction. Changes in external environmental conditions (e.g., drainage or nutrient input) alters the resilience landscape and a natural or human‐induced perturbation may push the system from a peat moss‐dominated state into a vascular plant state (right inset, red dotted arrow). To reverse this shift, favorable conditions (e.g., wet and nutrient‐poor) must be restored. Without achieved boundary conditions, active and continued intervention remains necessary to prevent relapse. Restoration actions like peat moss introduction or the disruption of vascular plants feedback can then initiate a state change. Fostering peat moss feedbacks aid to create a resilient peat moss‐dominated state. Main graph: Vertical colored arrows indicate the direction of change. Green and red balls indicate the change in environmental conditions and correspond with the resilience landscapes of the insets. Insets: Large colored ball = current system state (green shades for restored, red shades for degraded), gray filled basin = altered external physical conditions.

Feedbacks in *Sphagnum*‐dominated peatlands aid to buffer environmental change to a certain level (Robroek et al. 2009) or protect against catastrophic events, such as drought or fire (Albert‐Saiz et al. 2025; Blier‐Langdeau et al. 2022). In many peatlands, environmental conditions are rapidly altered resulting in an unstable system that can lead to peatland degradation (Figure 3). For example, a large proportion of European bogs are subjected to high atmospheric nitrogen deposition (Ackerman et al. 2019). Furthermore, hydrology is altered because of landscape‐scale drainage (Swindles et al. 2019). These environmental changes can lead to an unstable peat moss state, because conditions become adverse for peat moss growth (Figure 3). For example, under high atmospheric nitrogen deposition, the filter‐function of peat moss becomes inconsequential and can result in leaching of nitrogen to the rhizosphere (Lamers et al. 2000; Limpens et al. 2011). The higher nitrogen availability in the rhizosphere enhances the growth, competitive strength and dominance of vascular plants (Table 1, feedback 11) (Gunnarsson and Rydin 2000). Furthermore, once the peatland becomes too dry for the peat mosses because of drainage, the feedbacks that enhance the water availability for the mosses are insufficient to keep the mosses wet. For example, once water levels become too low for too long, the capillary feedback cannot maintain wet conditions at moss level and the albedo mechanism, the whitening of peat moss that reduces evaporative losses, will not be sufficient to protect the mosses from complete drying and can result in a die‐off (Table 1, feedback 3). In contrast, vascular plants are better able to germinate and thrive in drier conditions and lead to a transition into a vascular plant‐dominated state (Figures 2B and 3). The loss of peat moss‐generated feedbacks has been observed in ecosystem experiments. For example, in a whole‐ecosystem peatland warming experiment, peat moss emergent feedbacks that stimulated their competitive edge were lost because of extreme warming and desiccation (Norby et al. 2019) and resulted in vascular plants outcompeting peat moss via both belowground and aboveground growth (Malhotra et al. 2020; McPartland et al. 2020).

### 3.2 Vascular Plant Feedbacks

Vascular plants generally inhabit an environment that is drier and/or nutrient richer compared to peat mosses. While peat mosses are adapted to oligotrophic and acidic conditions and can outcompete vascular plants under such conditions, vascular plants can outcompete mosses under higher nutrient availability and/or lower water levels. Vascular plants generate emergent feedbacks that maintain drier and more nutrient‐rich conditions and thus reinforce an opposite state (Figures 2 and 3, Table 2). For example, high densities of vascular plants will shade peat mosses, releasing vascular plants from competition, which leads to more vascular plants that create more shade (Table 1, feedback 21) (Limpens et al. 2003; Malmer et al. 2003; Norby et al. 2023; Tomassen, Smolders, Limpens et al. 2004). Furthermore, a high density of vascular plants and trees increases evapotranspiration, creating drier and more nutrient‐rich conditions because of enhanced decomposition, which leads to less peat moss, releasing vascular plants from competition, which leads to more vascular plants that further enhance evapotranspiration (Table 2) (Gallagher et al. 2002). However, lower densities of vascular plants can be beneficial to peat moss growth by, for example, providing physical structure (Table 1, feedback 15) (Keightley et al. 2023; Malmer et al. 1994; Pouliot et al. 2011; Telgenkamp et al. 2025; Weber 1902).

**TABLE 2:** Feedbacks in vascular plant‐dominated systems.

Feedback name	Feedback type	Feedback description	Temporal scale and application			Operates under the following conditions		References
			Restoration potential	Practice	Temporal scale to create the feedback	Conditions	Spatial scale	
*Feedbacks that increase vascular plants' competitive edge and reduce the establishment of peat mosses*								
(20) Evapotranspiration (self‐amplifying)	Biophysical	High densities of vascular plants increase evapotranspiration, creating drier and more nutrient‐rich conditions leading to less peat moss, releasing vascular plants from competition, which leads to more vascular plants that further enhance transpiration. Lower densities can be beneficial to peat moss growth (see feedback 18).	Medium	Removing certain vascular plants; tree cutting (large scale often not feasible); maintaining a water table near the peat surface (saturation) to avoid tree establishment or achieve tree mortality	Years	All	dm^2^ to m^2^	Farrick and Price (2009); Fay and Lavoie (2009); Guêné‐Nanchen et al. (2017); Hirano et al. (2016); Rietkerk et al. (2004); Waddington et al. (2015)
(21) Shade (self‐amplifying)	Biophysical	High densities of vascular plants increase shade, which leads to less peat moss, releasing vascular plants from competition, which leads to more vascular plants that create more shade. An increase of vascular plants result in more litter production, which accumulates on top of peat mosses, creating shade and prevent growth (provided that litter mats are very thick and persistent).	Medium	Removing plants and/or the removing litter. Large‐scale mowing can be challenging	Years	All	m^2^	Limpens et al. (2003); Malmer et al. (2003); Norby et al. (2023); Tomassen, Smolders, Limpens et al. (2004); Guêné‐Nanchen et al. (2017)
(22) Radial oxygen loss	Biological	Vascular plants shunt oxygen in the waterlogged peat, which result in an oxygenized layer with enhanced decomposition and nutrient release, which stimulates the growth of vascular plants.	Medium	Removing plants, large‐scale removal can be challenging	Years	All	m^2^	Armstrong (1967); Lai et al. (2012)
(23) Nitrogen interception (self‐amplifying)	Biophysical	An increase of vascular plants and trees increases the interception of dry nitrogen deposition, which enhances the availability of nitrogen that enhances vascular plants and trees growth. Also, the presence of trees increases the presence of birds, which result in increased nutrient input, which stimulates plant and tree growth.	Medium	Removing vascular plants and/or the removal of trees. This can be challenging on a large scale	Years	All	m^2^ to ha	Gallagher et al. (2002); Tomassen et al. (2005)

*Note:* A prerequisite to establish *Sphagnum* feedbacks and disrupt vascular‐plants feedbacks is that the site is rewetted. The feedbacks are ordered according to their spatial scale (small to large). All feedbacks are self‐amplifying, where vascular plants promote conditions that further increase vascular plants' development until carrying capacity is reached or poor environmental conditions overwhelm the feedback. The literature list is not exhaustive. For a brief method description see caption Table 1.

## 4 Harnessing Emergent Feedbacks to Accelerate Peatland Restoration

Peatland protection and restoration have largely focused on approaches that assume the re‐creation of historical abiotic (pre‐degradation) conditions through rewetting (black arrows of the left inset in Figure 3). Moreover, such approaches often aim to return the ecosystem to its original state (e.g., Kreyling et al. 2021). While restoration successes have been obtained (Allan et al. 2023; Breton et al. 2026; Bruland and Richardson 2005; Convention on Wetlands 2021; González and Rochefort 2019), emergent feedbacks are not explicitly considered and applied at a large scale (feedback loop arrow in the left inset in Figure 3) (Convention on Wetlands 2021). Indeed, evidence shows that rewetting alone does not necessarily lead to the recovery of target vegetation (Allan et al. 2023; Kreyling et al. 2021). This implies that without addressing underlying feedbacks, restoration efforts may fail to shift systems out of an undesirable basin of attraction, even if water regimes are successfully restored (Figures 3 and 5).

### 4.1 Context‐Dependent Restoration

**FIGURE 4:**
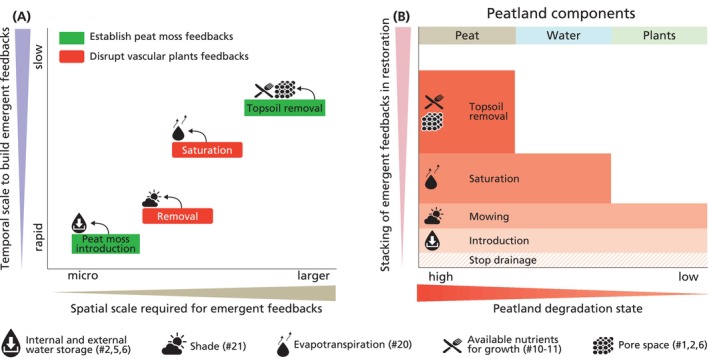
Conceptual overview of selected feedbacks that function at various temporal and spatial scales. (A) Peatland restoration requires establishing peat moss feedbacks while disrupting feedbacks that reinforce vascular plant dominance. (B) The amount of measures required for restoration depends on the degree of peatland degradation. As degradation increases, restoration becomes more complex and non‐linear, since peatland components, namely plants, water, and peat, are increasingly altered and harder to restore. Restoration should begin by ensuring base conditions (e.g., wetness, low nitrogen levels) that allow peat moss growth (see black arrows of the left inset in Figure 3). From there, measures like peat moss introduction and breaking of vascular plant feedbacks are needed as a perturbation for a state change (green arrow in Figure 3) and depends on the degradation state. (A) Green rectangles indicate actions to establish peat moss feedbacks and also disrupt vascular plant feedbacks. Red and rounded rectangles indicate actions to disrupt vascular plant feedbacks. The text provides examples of interventions, symbols denote feedbacks and # refers to feedbacks listed in Tables 1 and 2.

Rewetting degraded peatlands is often a key first step to restore peatlands. Next to this, we argue that restoration practitioners should take deliberate action to use feedbacks to accelerate peatland restoration through the establishment of emergent peat moss feedbacks and/or the disruption of vascular plant feedbacks (green arrow in Figures 3 and 4). Fostering feedbacks in restoration would be especially useful under moderate environmental stress that allows peat moss growth and where there is bistability (moderately harsh external environmental conditions in Figure 3). Bistability is a condition where, depending on the initial state, either a peat moss or a vascular plant state is stable under the same external environmental conditions (Scheffer et al. 2001; van der Velde et al. 2021). Under benign conditions, there is only one stable state that is dominated by peat mosses, while under harsh conditions vascular plants dominate. For restoration, this implies that harsh external environmental conditions need to be modified to at least intermediate levels (Figure 3).

Degraded peatlands each have a unique history of human‐caused problems with a specific state of degradation (Convention on Wetlands 2021). When a peatland is drained, degradation typically begins with the lowering of the water table and then the loss of wetland plants, followed by longer‐term changes in hydrology, and eventually the slow, often irreversible breakdown of the peat soil matrix (Figure 4). Consequently, as peat degradation increases, the number of required restoration interventions rises and the effort needed to achieve restoration goals increases non‐linearly (Rillig et al. 2024). Restoration practitioners thus need to define the degradation state of their system (chemical conditions, residual topography, hydrological setting, presence of invasive species) and set goals and suitable measures to restore emergent feedbacks (Suding et al. 2015). Once these moderately harsh base conditions are re‐created, interventions to re‐establish peat moss or disrupt vascular plant feedbacks can be relatively straightforward at a low degradation state (i.e., involving the plant component). In such cases, the introduction (or spread) of peat mosses can be sufficient to re‐establish internal and external water feedbacks (Table 1). When degradation is more severe (i.e., involving the plant and water components), additional measures may be needed to disrupt the vascular plant‐induced shade and transpiration feedbacks, such as removing vascular plants or cutting trees that is aided by blockage of shallow drains to reduce water loss and thereby promote peat moss establishment and growth (Table 1). In contrast, a degradation state where plant species composition, hydrology and water chemistry, and peat components are all heavily affected, restoration practitioners need to stack measures to restore peat moss dominance. For example, practitioners should first restore the porosity and remove accumulated soil nutrients with an expensive topsoil removal method (i.e., peat component), take measures to retain water in the system (i.e., water component), lower evapotranspiration through the removal of vascular plants or cutting trees when atmospheric N‐deposition is high, and introduce target peat moss species for albedo, acidification, peat buildup and internal and external water storage (i.e., plant component).

A key challenge in restoring emergent feedbacks is the wide variation among peat moss species in growth forms and habitat preferences (Clymo and Hayward 1982; Michaelis 2019). Peat moss‐driven feedbacks are not equally strong among all species. For example, there is large variation between species with respect to water retention capacity and in rates of growth and decomposition. These species‐specific characteristics will influence how and to what extent peat mosses regulate plant succession and associated peatland functions and can be used in ecological restoration (Chirino et al. 2006; Granath et al. 2010; Grau‐Andrés et al. 2022; Johnson and Damman 1991; Rice et al. 2008). Closely related species tend to exhibit similar trait values and some generalizations can be made (Piatkowski and Shaw 2019). For example, species of the subgenus *Acutifolia* are mostly hummock‐forming with a low rate of decomposition and a high water‐retention capacity (Bengtsson et al. 2016, 2020). *Acutifolia* represent a strategy of resource conservation (Mazziotta et al. 2019; slow growth, sensu Reich 2014), which can make them less successful in early stages of peatland development but exceptions exist (Chirino et al. 2006). In contrast, fast‐growing and rapidly decomposing hollow‐dwelling species follow a strategy of resource acquisition (Mazziotta et al. 2019), as found in the subgenus *Cuspidata*. The subgenus *Sphagnum* contains several intermediate species within this fast‐to‐slow continuum. Recent work has shown that a low cover of vascular plants that grows in moss lawns dominated with species of the subgenus *Cuspidata* can facilitate the encroachment of species from the subgenus *Acutifolia* (Telgenkamp et al. 2025). Such facilitative interactions can enhance the recovery of feedbacks that promote the return of a functioning and self‐regulating acrotelm.

### 4.2 Challenges for the Initiation of Peat Moss Feedbacks

**FIGURE 5:**
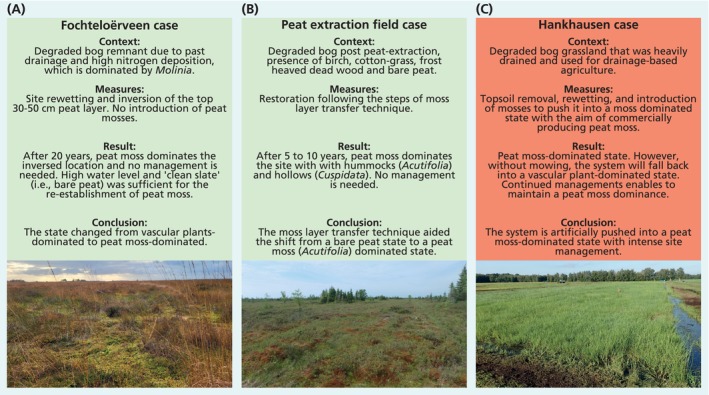
Overview of three restoration efforts and the outcomes. (A) Fochteloërveen in The Netherlands, (B) Sainte‐Marguerite‐Marie peatland in Canada, and (C) Hankhausen in Germany; note peat mosses are prevalent under the tall growing grass. Photo credits: (A and C) R.J.M. Temmink and (B) Peatland Ecology Research Group.

The importance of peat mosses in generating emergent feedbacks that enhance ecosystem functioning is evident (Table 1). In some cases, restoration successfully achieved a state change from a vascular plant‐dominated to a peat moss‐dominated state, while in other instances continued management is needed to support peat moss dominance (Figure 5). Natural, spontaneous recolonization by peat moss is often slow in severely degraded peatlands, such as agricultural peat soils or former peat extracted sites when rewetting is the only restoration measure applied. Consequently, peat mosses have to be actively reintroduced to accelerate peatland restoration. The reintroduction of mosses, combined with straw mulch application and a diverse mix of diaspores (including peat moss and *Polytrichum* species), is an effective and cost‐efficient strategy for restoring extensive areas, ranging from tens to hundreds of hectares (Breton et al. 2026; Rochefort et al. 2003). For smaller areas, planting a high density and/or large patch size of mosses (> 15 cm in diameter) will yield good results, although costly in material supply and human resources (Gage et al. 2024; Robroek et al. 2009; Smolders et al. 2003). However, in heavily degraded landscapes, the required donor material is frequently limited and presents challenges for ecological restoration.

In Western Central Europe, a hotspot of bog degradation, peat mosses in bog remnants are typically unavailable for restoration either because of scarcity or strict legal protection. This creates a restoration challenge: accelerating the restoration of peat‐moss‐dominated peatlands through emergent feedbacks requires donor material, yet local sources are scarce or unavailable. For example, 1 m^2^ of vegetation is needed to restore 10–15 m^2^ with the moss layer transfer technique (Breton et al. 2026). Recent research suggests that paludiculture might offer a solution. Paludiculture is agriculture on wet peatlands, in which wetland plants are cultivated for commercial use (Temmink et al. 2026; Wichtmann et al. 2016). For example, peat moss paludiculture can serve as a donor production site in which peat moss can rapidly grow under optimal water and nutrient management and be harvested to be used as founder material in bog restoration (Gaudig et al. 2018; Hugron and Rochefort 2018; Pouliot et al. 2015; Temmink et al. 2024; Wichmann et al. 2020).

Terrestrialization is another challenge for restoration in certain settings where large human‐made pools exist. In bog remnants, managers mainly target hydrology and atmospheric nitrogen deposition mitigation, but often also face challenges with the terrestrialization of open water (Temmink, Cruijsen, Smolders et al. 2021). When such human‐made pools are too large, strongly colored by humic acids or have insufficient carbon dioxide in the water layer, terrestrialization does not occur (Kreyling et al. 2021; Patberg et al. 2013; Smolders et al. 2003; Temmink, Cruijsen, Smolders et al. 2021; Weber 1902). These pools are also characterized by two alternative states: open water or a carpet of floating peat moss. The colonization is strongly dependent on levels of carbon dioxide and the fetch (Patberg et al. 2013). Recent research showed that the use of biodegradable structures that mimic a floating peat moss carpet in open water can kickstart moss establishment in an open water state (Temmink, Cruijsen, Smolders et al. 2021). Such mimicry can also be used to reduce the amount of donor material required in restoration, as has been done for cord grasses, seagrasses, and bivalves (Temmink, Angelini, Fivash et al. 2021; Temmink et al. 2020; van der Heide et al. 2021).

## 5 Broader Implications and Pathways Forward

We synthesized how current knowledge of emergent feedbacks can be applied to accelerate the restoration of peat moss‐dominated peatlands. However, questions remain on how practitioners can optimize donor material and spreading design to faster adapt to global change (Fivash et al. 2022; Michaels et al. 2023). One way forward is the cultivation of the moss donor material in paludiculture sites or micropropagated peat moss that is first cultivated and then spread on the target site (Caporn et al. 2018; Gaudig et al. 2018; Keightley et al. 2024; Pouliot et al. 2015; Temmink et al. 2024). Alternatively, natural peat moss colonization from spores can be promoted. Although knowledge is limited and the process is slow and difficult, it can also help restoration of peatlands with vegetation but without target peat moss species, such as peatlands drained for forestry. Germination of peat moss spores is limited by low nutrient availability and germination is predicted to increase with a consistently moist peat surface layer in combination with litter from vascular plants and potentially phosphorus addition (Sundberg and Rydin 2002). To overcome these bottlenecks, mimicry of emergent feedbacks might aid in the establishment from spores, which has proven successful in enhancing transplant survival or establishment from seed or larvae in coastal restoration (Fivash et al. 2021; Temmink, Angelini, Fivash et al. 2021; Temmink et al. 2020). Furthermore, we need a better understanding of interactions among the small scale feedbacks discussed here and the spatially (and organizationally) larger feedbacks, such as the feedbacks in hummock‐hollow patterns (Couwenberg et al. 2022; Waddington et al. 2015). Lastly, we call scientists to experimentally test how emergent feedbacks can be harnessed in their respective peatland ecosystems at various spatial and temporal scales.

Changes in soil microbial communities, along with organic inputs from litter and root exudates, create microbial and chemical legacies that likely differ between peat moss– and vascular plant–dominated peatlands (Defrenne et al. 2023; Palozzi and Lindo 2017a, 2017b). An important yet often overlooked factor in peatland restoration is the role that these soil microbial and chemical legacies, which are important mechanisms driving plant–soil feedbacks, play in modulating restoration success (Andersen et al. 2013; Delory et al. 2024). From a restoration perspective, soil legacies are important because they can shape competitive dynamics and regulate key ecosystem processes (Kardol et al. 2007; Pugnaire et al. 2019). It may either reinforce the dominance of certain species (positive feedback) or prevent dominant species from excluding others, thereby supporting the persistence of sub‐dominant species (negative feedback) (Maron et al. 2011; Mommer et al. 2018). In particular, we lack understanding of how soil microbial and chemical legacies contribute to peatland community dynamics and how their associated mechanisms respond to global environmental changes, such as climate change and nutrient pollution (Bragazza et al. 2013; Jassey et al. 2018; Shao et al. 2023; Živković et al. 2025). Despite methodological challenges in peat soils, including a high organic matter content that makes root extraction and plant removal after soil conditioning difficult, plant–soil feedbacks are essential for advancing our understanding of ecological processes in peatlands and for improving restoration strategies.

For successful restoration of peat moss‐dominated ecosystems, it is crucial to monitor the effects of the implemented restoration measures to ensure that the intended emergent feedbacks are established at broad spatial scales. This can inform decision‐making on potential additional interventions, when needed, but also build on the existing understanding of emergent feedbacks for peat moss‐dominated ecosystems (Table 1). Furthermore, emergent feedback monitoring and measurements in restoration settings can enable mechanistic modeling of peatland restoration and help project the carbon cycle outcomes of peatland restoration (Nugent et al. 2019). As these emergent feedbacks operate across a range of spatiotemporal scales, the monitoring strategy should be appropriate to cover such scales. Long‐term monitoring is especially required, because peatlands develop slowly and as a consequence projects may appear as a failure in the short term, but as a success in the long term (González and Rochefort 2014). Considering these requirements, an effective monitoring strategy can be developed around the use of remote sensing technology, including proximal remote sensing, to airborne and satellite‐based approaches (Kooistra et al. 2024; Räsänen et al. 2025; Salko et al. 2023). However, as peat moss species identity can be essential for feedbacks to develop, current remote sensing approaches are not enough and assessment through reoccurring field visits is required. Ideally, these complementary monitoring approaches in the field are set up to achieve maximum synergy, e.g., targeting a field visit to a specific site after remote sensing detected unusual patterns (Szantoi et al. 2016).

The framework presented here is applicable in feedback‐driven ecosystems worldwide, such as salt marshes, dunes, seagrass, mangroves, arid drylands and coral reefs. We argue that emergent feedbacks should not only be harnessed to increase restoration success (Maxwell et al. 2016; Silliman et al. 2015; Temmink et al. 2020; van Katwijk et al. 2016), but should also be linked and tailored to the degradation state of an ecosystem. Therefore, this conceptual framework of emergent feedbacks has the potential to advance restoration science of feedback‐driven ecosystems. As feedback‐driven systems support many ecosystem services (Breton et al. 2026; Maxwell et al. 2016; Temmerman et al. 2013; Temmink et al. 2022), their restoration can facilitate humanity to reach targets set by the Paris Climate Agreement, Kunming‐Montreal Global Biodiversity Framekwork, EU Nature Restoration Regulation and the United Nations Decade on Ecosystem Restoration.

## Author Contributions

**Ralph J. M. Temmink:** conceptualization, visualization, writing – original draft, writing – review and editing. **Benjamin M. Delory:** writing – review and editing. **Max Rietkerk:** visualization, writing – review and editing. **Alfons J. P. Smolders:** writing – review and editing. **Leon P. M. Lamers:** writing – review and editing. **Avni Malhotra:** writing – review and editing. **Line Rochefort:** writing – review and editing. **Gustaf Granath:** writing – review and editing. **John Couwenberg:** writing – review and editing. **Gerbrand Koren:** writing – review and editing. **Bjorn J. M. Robroek:** writing – review and editing.

## Funding

R.J.M.T. was funded by NWO/ENW Veni grant 232.039 and NWO‐AES grant 21761. L.R. was funded by Natural Sciences and Engineering Research Council of Canada (NSERC Discovery grant, no. 138097‐2012, RGPIN‐2018‐06080, RGPIN‐2024‐03832). The research of M.R. is supported by the European Research Council (ERC‐Synergy project RESILIENCE, proposal nr. 101071417) and by the Dutch Research Council (NWO “Resilience in complex systems through adaptive spatial pattern formation,” project nr. OCENW.M20.169). This work was conducted as part of the EMBRACER program, the Earth System Feedback Research Centre, and was financially supported by the SUMMIT program of the Dutch Research Council (NWO). A.M. was supported by the Swiss National Science Foundation (project 200021_215214) and an Early Career Award by the U.S. Department of Energy, Office of Science, Biological and Environmental Research. B.J.M.R. was supported through a 2020–2021 Biodiversa+ and Water JPI joint call for research projects, under the BiodivRestore ERA‐NET Cofund (GA N° 101003777), with financial support from the Ministry of LNV (The Netherlands). G.G. was funded by FORMAS (grant no 2022‐02106).

## Conflicts of Interest

The authors declare no conflicts of interest.

## Data Availability

No data was used for the research described in the article.
